# Dimethyl 9-benzyl-3-cyano-9*H*-pyrrolo[1,2-*a*]benzimidazole-1,2-dicarboxyl­ate

**DOI:** 10.1107/S1600536809048867

**Published:** 2009-11-21

**Authors:** Wei-Jin Gu, Yu-Liang Jiang, Bing-Xiang Wang

**Affiliations:** a Department of Applied Chemistry, Nanjing Normal University, Nanjing 210097, People’s Republic of China

## Abstract

The title compound, C_22_H_17_N_3_O_4_, was prepared through 1,3-dipolar cyclo­addition: the dihedral angle between the benzimidazole and benzene rings is 80.93 (6)°. The crystal structure is stabilized by weak π–π inter­actions between the planar pyrrolobenzimidazole rings (r.m.s. deviation = 0.0293 Å) of neighbouring mol­ecules, forming chains along the *c* axis. The perpendicular distance is 3.47 (2) Å and the centroid–centroid distances are in the range of 3.590 (3)–3.944 (3) Å.

## Related literature

For the use of 1,3-dipolar cyclo­addition reactions of azomethine ylides in the construction of five-membered nitro­gen heteroaromatic ring systems, see: Berry *et al.* (2007 ▶). For the applications of nitro­gen heteroaromatic ring systems, see: Ansari & Lal (2009 ▶); Shen *et al.* (2006 ▶, 2008 ▶); Zhang *et al.* (2009 ▶). For the synthesis, see: Wang *et al.* (2000 ▶).
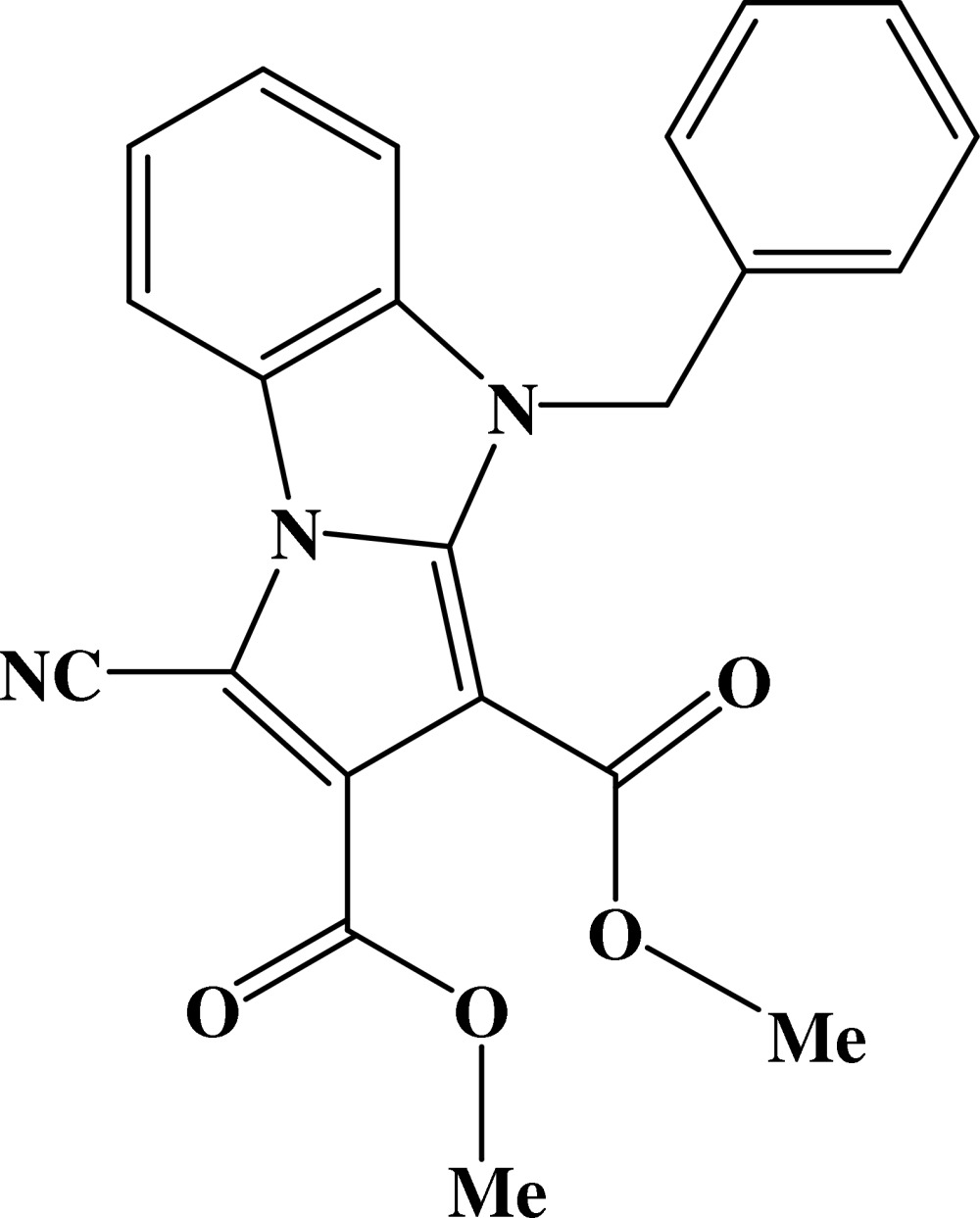


## Experimental

### 

#### Crystal data

C_22_H_17_N_3_O_4_
*M* *_r_* = 387.39
Monoclinic, 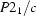

*a* = 9.8681 (15) Å
*b* = 24.766 (2) Å
*c* = 7.6551 (11) Å
β = 91.973 (3)°
*V* = 1869.7 (4) Å^3^
*Z* = 4
Mo *K*α radiation
μ = 0.10 mm^−1^
*T* = 291 K
0.26 × 0.22 × 0.20 mm

#### Data collection

Bruker SMART APEX CCD diffractometer
Absorption correction: multi-scan (*SADABS*; Bruker, 2000 ▶) *T*_min_ = 0.97, *T*_max_ = 0.98
13898 measured reflections
3615 independent reflections
2699 reflections with *I* > 2σ(*I*)
*R*_int_ = 0.057

#### Refinement

*R*[*F* ^2^ > 2σ(*F* ^2^)] = 0.056
*wR*(*F* ^2^) = 0.125
*S* = 1.03
3615 reflections
264 parameters
H-atom parameters constrained
Δρ_max_ = 0.19 e Å^−3^
Δρ_min_ = −0.19 e Å^−3^

### 

Data collection: *SMART* (Bruker, 2000 ▶); cell refinement: *SAINT* (Bruker, 2000 ▶); data reduction: *SAINT*; program(s) used to solve structure: *SHELXTL* (Sheldrick, 2008 ▶); program(s) used to refine structure: *SHELXTL*; molecular graphics: *SHELXTL*; software used to prepare material for publication: *SHELXTL*.

## Supplementary Material

Crystal structure: contains datablocks global, I. DOI: 10.1107/S1600536809048867/zq2016sup1.cif

Structure factors: contains datablocks I. DOI: 10.1107/S1600536809048867/zq2016Isup2.hkl

Additional supplementary materials:  crystallographic information; 3D view; checkCIF report

## Figures and Tables

**Fig. 1.**
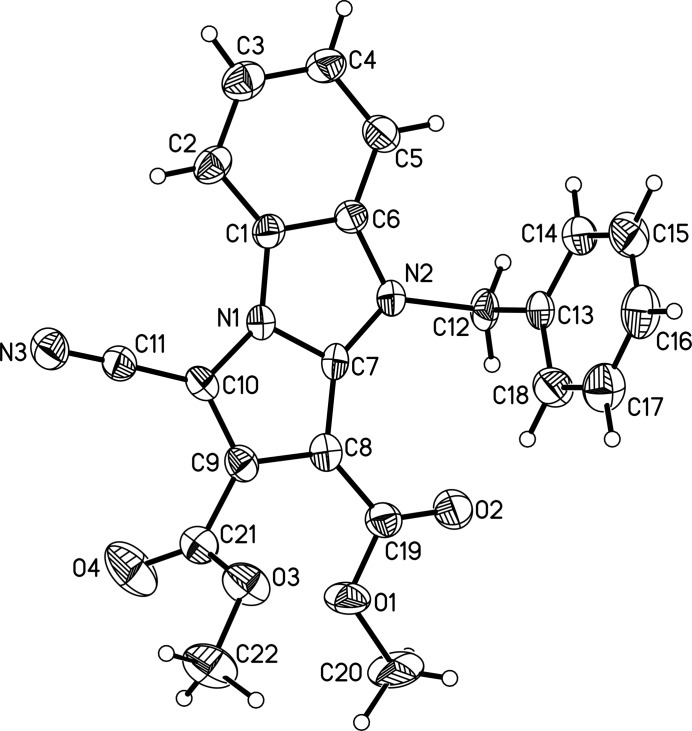
A view of the title compound showing the atom-numbering scheme and displacement ellipsoids drawn at 50% probability level.

**Fig. 2.**
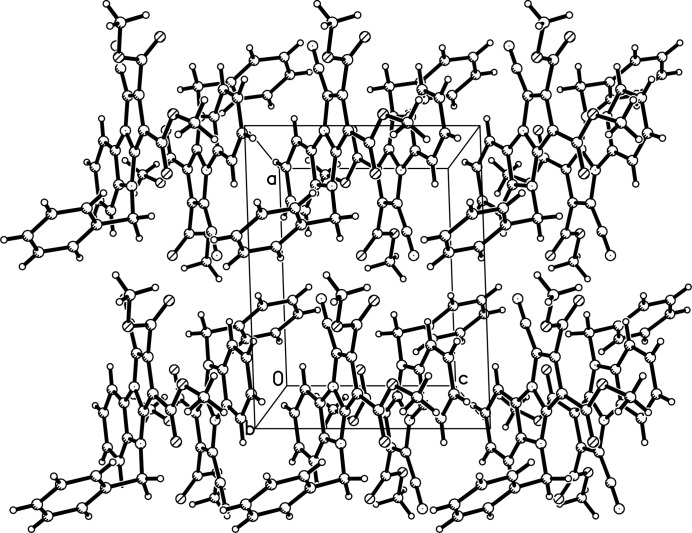
A view of the packing down b axis.

**Fig. 3.**
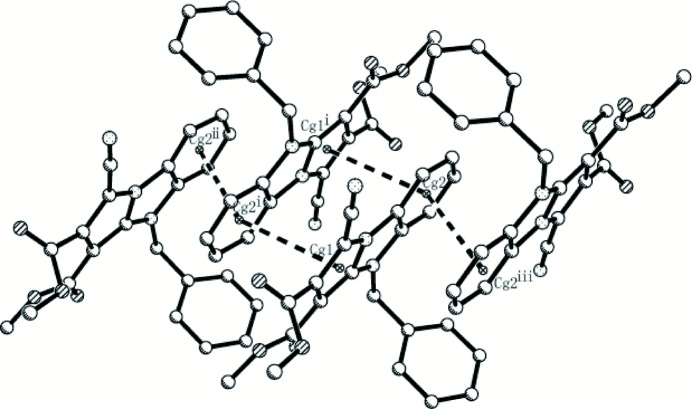
A view of the one-dimensional chains down b axis. Dashed lines indicate weak π–π interaction and all H atoms have been omitted for clarity (i: 2 - x, 2 - y, 2 - z; ii: x, y, 1 + z; iii: 2 - x, 2 - y, 1 - z).

## supplementary crystallographic information

### Comment

1,3-Dipolar cycloadditions of azomethine ylides are one of the most powerful methods for the construction of five membered nitrogen heteroaromatic ring systems, both inter and intramolecularly (Berry *et al.*, 2007). Nitrogen heteroaromatic ring systems, such as benzimidazoles and indolizines, can be utilized as not only a wide variety of biologically active and medicinally significant compounds (Zhang *et al.*, 2009; Ansari *et al.*, 2009), but also organic fluorescence probes (Shen *et al.*, 2006; Shen *et al.*, 2008). In our continuing studies in organic fluorescence probes, we synthesized the title compound (I), dimethyl 4-benzyl-1-cyano-4*H*-pyrrolo[1, 2 - a] benzimidazole-2,3-dicarboxylate, C_22_H_17_N_3_O_4_.

The crystal structure of (I) reveals that all the bond lengths and angles have normal values. There is one title compound molecule per asymmetric unit. Each molecule contains one pyrrolo-benzimidazole ring A and one benzyl ring B (Fig 1). The rings A and B are almost perpendicular, making a dihedral angle of 80.93 (6)°.

In the crystal structure there are weak π–π interactions between the planar pyrrolo-benzimidazole rings (r.m.s. deviation of 0.0293 Å) of neighbouring molecules. The perpendicular distance is 3.47 (2) Å and the distances *Cg*1—*Cg*2^i^ and *Cg*2—*Cg*2^iii^ are 3.590 (3) and 3.944 (3) Å, respectively. (*Cg*1 is the center of ring C7/C8/C9/C10/N1 and *Cg*2 is the center of ring C1/C2/C3/C4/C5/C6; i: 2 - *x*, 2 - *y*, 2 - *z*; iii: 2 - *x*, 2 - *y*, 1 - *z*). Through the π–π interactions one-dimensional chains are formed along *c* axis.

### Experimental

Dimethyl 4-benzyl-1-cyano-4*H*-pyrrolo[1,2-a] benzimidazole-2,3-dicarboxylate was prepared through 1,3-dipolar cycloaddition according to a procedure described in the literature (Wang *et al.*, 2000). Colorless crystals were obtained by recrystallization from a dichloromethane solution at room temperature.

### Refinement

The H atoms were positioned geometrically and refined using a riding model (including free rotation about the ethanol C—C bond), with C—H = 0.93–0.97 Å and with *U*_iso_(H) = 1.2 (1.5 for methyl groups) times *U*_eq_(C).

### Crystal data

**Table:**

C_22_H_17_N_3_O_4_	*F*(000) = 808
*M**_r_* = 387.39	*D*_x_ = 1.376 Mg m^−^^3^
Monoclinic, *P*2_1_/*c*	Mo *K*α radiation, λ = 0.71073 Å
Hall symbol: -P 2ybc	Cell parameters from 1791 reflections
*a* = 9.8681 (15) Å	θ = 2.6–25.2°
*b* = 24.766 (2) Å	µ = 0.10 mm^−^^1^
*c* = 7.6551 (11) Å	*T* = 291 K
β = 91.973 (3)°	Block, colorless
*V* = 1869.7 (4) Å^3^	0.26 × 0.22 × 0.20 mm
*Z* = 4	

### Data collection

**Table:**

Bruker SMART APEX CCD diffractometer	3615 independent reflections
Radiation source: sealed tube	2699 reflections with *I* > 2σ(*I*)
graphite	*R*_int_ = 0.057
phi and ω scans	θ_max_ = 26.0°, θ_min_ = 1.6°
Absorption correction: multi-scan (*SADABS*; Bruker, 2000)	*h* = −12→12
*T*_min_ = 0.97, *T*_max_ = 0.98	*k* = −26→30
13898 measured reflections	*l* = −9→9

### Refinement

**Table:**

Refinement on *F*^2^	Primary atom site location: structure-invariant direct methods
Least-squares matrix: full	Secondary atom site location: difference Fourier map
*R*[*F*^2^ > 2σ(*F*^2^)] = 0.056	Hydrogen site location: inferred from neighbouring sites
*wR*(*F*^2^) = 0.125	H-atom parameters constrained
*S* = 1.02	*w* = 1/[σ^2^(*F*_o_^2^) + (0.05*P*)^2^ + 0.66*P*] where *P* = (*F*_o_^2^ + 2*F*_c_^2^)/3
3615 reflections	(Δ/σ)_max_ < 0.001
264 parameters	Δρ_max_ = 0.19 e Å^−^^3^
0 restraints	Δρ_min_ = −0.19 e Å^−^^3^

### Special details

**Table:**

Experimental. 1*H*-NMR (CDCl_3_, 400 MHz) δ: 3.81 (s, 3H, –COOCH_3_), 4.00 (s, 3H, –COOCH_3_), 5.92 (s, 2H, –CH_2_Ph), 7.20–7.41 (m, 8H, ArH), 8.07 (d, 1H, J = 7.9 Hz, ArH)
Geometry. All e.s.d.'s (except the e.s.d. in the dihedral angle between two l.s. planes) are estimated using the full covariance matrix. The cell e.s.d.'s are taken into account individually in the estimation of e.s.d.'s in distances, angles and torsion angles; correlations between e.s.d.'s in cell parameters are only used when they are defined by crystal symmetry. An approximate (isotropic) treatment of cell e.s.d.'s is used for estimating e.s.d.'s involving l.s. planes. Least-squares planes (*x*,*y*,*z* in crystal coordinates) and deviations from them (* indicates atom used to define plane)-0.0001(0.0049) *x* + 11.1526(0.0083)*y* + 6.8309(0.0017) *z* = 16.2473 (0.0105)* 0.0370 (0.0017) C1 * 0.0187 (0.0017) C2 * -0.0329 (0.0017) C3 * -0.0312 (0.0019) C4 * -0.0132 (0.0017) C5 * 0.0115 (0.0017) C6 * 0.0056 (0.0017) C7 * -0.0396 (0.0016) C8 * -0.0420 (0.0016) C9 * 0.0128 (0.0016) C10 * 0.0392 (0.0015) N1 * 0.0341 (0.0015) N2Rms deviation of fitted atoms = 0.02937.9901(0.0063)*x* + 10.7417(0.0233)*y* - 3.2379(0.0071)*z* = 12.8495 (0.0190)Angle to previous plane (with approximate e.s.d.) = 80.93 (0.06)* 0.0109 (0.0016) C13 * -0.0131 (0.0017) C14 * 0.0058 (0.0018) C15 * 0.0036 (0.0019) C16 * -0.0055 (0.0019) C17 * -0.0016 (0.0017) C18Rms deviation of fitted atoms = 0.0078
Refinement. Refinement of *F*^2^ against ALL reflections. The weighted *R*-factor *wR* and goodness of fit *S* are based on *F*^2^, conventional *R*-factors *R* are based on *F*, with *F* set to zero for negative *F*^2^. The threshold expression of *F*^2^ > σ(*F*^2^) is used only for calculating *R*-factors(gt) *etc*. and is not relevant to the choice of reflections for refinement. *R*-factors based on *F*^2^ are statistically about twice as large as those based on *F*, and *R*- factors based on ALL data will be even larger.

### Fractional atomic coordinates and isotropic or equivalent isotropic displacement parameters (Å^2^)

**Table:**

	*x*	*y*	*z*	*U*_iso_*/*U*_eq_	
C1	1.0019 (2)	1.00932 (8)	0.7360 (3)	0.0272 (4)	
C2	1.0476 (2)	1.05587 (9)	0.6574 (3)	0.0342 (5)	
H2	1.1394	1.0634	0.6481	0.041*	
C3	0.9480 (3)	1.09034 (10)	0.5935 (3)	0.0403 (6)	
H3	0.9727	1.1219	0.5367	0.048*	
C4	0.8095 (2)	1.07867 (9)	0.6128 (3)	0.0387 (6)	
H4	0.7450	1.1033	0.5710	0.046*	
C5	0.7662 (2)	1.03148 (10)	0.6925 (3)	0.0359 (5)	
H5	0.6746	1.0239	0.7042	0.043*	
C6	0.8651 (2)	0.99690 (9)	0.7526 (3)	0.0283 (5)	
C7	0.9829 (2)	0.92732 (8)	0.8653 (3)	0.0252 (4)	
C8	1.0572 (2)	0.88306 (9)	0.9310 (3)	0.0283 (5)	
C9	1.1957 (2)	0.89723 (9)	0.9075 (3)	0.0301 (5)	
C10	1.2040 (2)	0.94775 (9)	0.8330 (3)	0.0288 (5)	
C11	1.3184 (2)	0.97791 (9)	0.7903 (3)	0.0333 (5)	
C12	0.7257 (2)	0.91558 (10)	0.8516 (3)	0.0326 (5)	
H12A	0.6509	0.9402	0.8692	0.039*	
H12B	0.7325	0.8913	0.9509	0.039*	
C13	0.69949 (19)	0.88336 (9)	0.6848 (3)	0.0292 (5)	
C14	0.6168 (2)	0.90361 (10)	0.5555 (3)	0.0394 (6)	
H14	0.5747	0.9368	0.5706	0.047*	
C15	0.5946 (3)	0.87514 (11)	0.4004 (3)	0.0463 (7)	
H15	0.5403	0.8898	0.3108	0.056*	
C16	0.6539 (3)	0.82453 (12)	0.3795 (3)	0.0493 (7)	
H16	0.6382	0.8055	0.2761	0.059*	
C17	0.7340 (3)	0.80288 (12)	0.5082 (3)	0.0466 (6)	
H17	0.7729	0.7690	0.4944	0.056*	
C18	0.7576 (2)	0.83240 (10)	0.6629 (3)	0.0411 (6)	
H18	0.8125	0.8179	0.7521	0.049*	
C19	1.0032 (2)	0.83746 (9)	1.0316 (3)	0.0336 (5)	
C20	1.0629 (3)	0.76423 (12)	1.2133 (4)	0.0577 (8)	
H20A	1.0048	0.7778	1.3013	0.087*	
H20B	1.1420	0.7482	1.2682	0.087*	
H20C	1.0150	0.7376	1.1441	0.087*	
C21	1.3202 (2)	0.86405 (10)	0.9391 (3)	0.0376 (5)	
C22	1.4343 (3)	0.78357 (12)	0.8747 (4)	0.0528 (7)	
H22A	1.5076	0.7961	0.8061	0.079*	
H22B	1.4102	0.7475	0.8400	0.079*	
H22C	1.4619	0.7838	0.9962	0.079*	
N1	1.07206 (16)	0.96508 (7)	0.8086 (2)	0.0243 (4)	
N2	0.85368 (17)	0.94658 (7)	0.8381 (2)	0.0278 (4)	
N3	1.41453 (19)	1.00265 (9)	0.7614 (3)	0.0411 (5)	
O1	1.10343 (16)	0.80841 (7)	1.1015 (2)	0.0403 (4)	
O2	0.88636 (17)	0.82715 (7)	1.0552 (2)	0.0449 (5)	
O3	1.31742 (17)	0.81903 (7)	0.8471 (2)	0.0451 (5)	
O4	1.4114 (2)	0.87751 (9)	1.0281 (3)	0.0648 (6)	

### Atomic displacement parameters (Å^2^)

**Table:**

	*U*^11^	*U*^22^	*U*^33^	*U*^12^	*U*^13^	*U*^23^
C1	0.0323 (11)	0.0244 (11)	0.0247 (10)	−0.0022 (9)	−0.0037 (8)	−0.0041 (8)
C2	0.0466 (13)	0.0273 (12)	0.0285 (11)	−0.0057 (10)	0.0002 (10)	−0.0019 (9)
C3	0.0513 (15)	0.0310 (13)	0.0385 (13)	0.0007 (11)	0.0013 (11)	−0.0045 (10)
C4	0.0433 (14)	0.0249 (12)	0.0473 (14)	0.0040 (10)	−0.0059 (11)	−0.0014 (10)
C5	0.0323 (12)	0.0352 (13)	0.0399 (13)	0.0041 (10)	−0.0055 (10)	−0.0037 (10)
C6	0.0274 (10)	0.0230 (11)	0.0343 (11)	0.0022 (9)	−0.0014 (9)	−0.0041 (9)
C7	0.0232 (10)	0.0269 (11)	0.0254 (10)	−0.0068 (8)	0.0006 (8)	−0.0034 (8)
C8	0.0264 (10)	0.0329 (12)	0.0257 (10)	−0.0025 (9)	0.0022 (8)	−0.0034 (9)
C9	0.0301 (11)	0.0342 (13)	0.0255 (10)	−0.0010 (9)	−0.0045 (9)	−0.0033 (9)
C10	0.0238 (10)	0.0337 (13)	0.0284 (10)	0.0024 (9)	−0.0040 (8)	−0.0013 (9)
C11	0.0260 (11)	0.0289 (12)	0.0448 (13)	0.0028 (9)	−0.0003 (10)	−0.0014 (10)
C12	0.0189 (10)	0.0365 (13)	0.0428 (13)	−0.0071 (9)	0.0074 (9)	−0.0026 (10)
C13	0.0175 (9)	0.0341 (12)	0.0361 (12)	−0.0056 (9)	0.0054 (8)	0.0021 (9)
C14	0.0381 (13)	0.0348 (13)	0.0444 (13)	−0.0044 (10)	−0.0116 (10)	−0.0010 (11)
C15	0.0522 (15)	0.0540 (17)	0.0316 (13)	−0.0098 (13)	−0.0143 (11)	0.0006 (11)
C16	0.0535 (16)	0.0536 (17)	0.0406 (14)	−0.0132 (13)	−0.0033 (12)	−0.0178 (12)
C17	0.0485 (15)	0.0460 (15)	0.0457 (15)	−0.0021 (12)	0.0038 (12)	−0.0133 (12)
C18	0.0398 (13)	0.0361 (14)	0.0466 (14)	0.0050 (11)	−0.0073 (11)	−0.0077 (11)
C19	0.0377 (13)	0.0303 (13)	0.0321 (12)	0.0005 (10)	−0.0074 (10)	−0.0015 (9)
C20	0.076 (2)	0.0544 (18)	0.0425 (15)	0.0009 (15)	0.0044 (14)	0.0349 (13)
C21	0.0348 (12)	0.0373 (14)	0.0403 (13)	0.0018 (10)	−0.0031 (10)	0.0009 (11)
C22	0.0480 (15)	0.0571 (18)	0.0535 (16)	0.0282 (13)	0.0064 (13)	−0.0133 (13)
N1	0.0211 (8)	0.0270 (9)	0.0249 (9)	−0.0030 (7)	0.0014 (7)	−0.0028 (7)
N2	0.0221 (8)	0.0295 (10)	0.0319 (9)	−0.0045 (7)	0.0000 (7)	−0.0004 (7)
N3	0.0303 (10)	0.0528 (14)	0.0404 (11)	−0.0052 (10)	0.0049 (8)	0.0129 (10)
O1	0.0418 (9)	0.0435 (10)	0.0356 (9)	0.0077 (8)	0.0030 (7)	0.0184 (7)
O2	0.0395 (10)	0.0547 (11)	0.0399 (9)	−0.0098 (8)	−0.0049 (7)	0.0225 (8)
O3	0.0467 (10)	0.0461 (11)	0.0422 (9)	0.0157 (8)	−0.0041 (8)	−0.0152 (8)
O4	0.0576 (12)	0.0899 (16)	0.0452 (11)	0.0140 (11)	−0.0211 (10)	−0.0184 (11)

### Geometric parameters (Å, °)

**Table:**

C1—C2	1.384 (3)	C12—H12B	0.9700
C1—C6	1.394 (3)	C13—C14	1.357 (3)
C1—N1	1.401 (3)	C13—C18	1.398 (3)
C2—C3	1.379 (3)	C14—C15	1.392 (3)
C2—H2	0.9300	C14—H14	0.9300
C3—C4	1.410 (4)	C15—C16	1.395 (4)
C3—H3	0.9300	C15—H15	0.9300
C4—C5	1.392 (3)	C16—C17	1.352 (4)
C4—H4	0.9300	C16—H16	0.9300
C5—C6	1.366 (3)	C17—C18	1.405 (3)
C5—H5	0.9300	C17—H17	0.9300
C6—N2	1.414 (3)	C18—H18	0.9300
C7—N1	1.365 (3)	C19—O2	1.201 (3)
C7—N2	1.371 (3)	C19—O1	1.321 (3)
C7—C8	1.402 (3)	C20—O1	1.454 (3)
C8—C9	1.429 (3)	C20—H20A	0.9600
C8—C19	1.477 (3)	C20—H20B	0.9600
C9—C10	1.378 (3)	C20—H20C	0.9600
C9—C21	1.491 (3)	C21—O4	1.159 (3)
C10—N1	1.378 (3)	C21—O3	1.318 (3)
C10—C11	1.401 (3)	C22—O3	1.459 (3)
C11—N3	1.157 (3)	C22—H22A	0.9600
C12—N2	1.485 (3)	C22—H22B	0.9600
C12—C13	1.520 (3)	C22—H22C	0.9600
C12—H12A	0.9700		
			
C2—C1—C6	123.7 (2)	C13—C14—H14	119.7
C2—C1—N1	131.3 (2)	C15—C14—H14	119.7
C6—C1—N1	104.97 (17)	C14—C15—C16	119.9 (2)
C3—C2—C1	115.5 (2)	C14—C15—H15	120.0
C3—C2—H2	122.2	C16—C15—H15	120.0
C1—C2—H2	122.2	C17—C16—C15	120.7 (2)
C2—C3—C4	121.2 (2)	C17—C16—H16	119.7
C2—C3—H3	119.4	C15—C16—H16	119.7
C4—C3—H3	119.4	C16—C17—C18	118.9 (3)
C5—C4—C3	122.1 (2)	C16—C17—H17	120.5
C5—C4—H4	118.9	C18—C17—H17	120.5
C3—C4—H4	118.9	C13—C18—C17	121.0 (2)
C6—C5—C4	116.5 (2)	C13—C18—H18	119.5
C6—C5—H5	121.7	C17—C18—H18	119.5
C4—C5—H5	121.7	O2—C19—O1	122.2 (2)
C5—C6—C1	120.9 (2)	O2—C19—C8	127.4 (2)
C5—C6—N2	129.9 (2)	O1—C19—C8	110.4 (2)
C1—C6—N2	109.22 (17)	O1—C20—H20A	109.5
N1—C7—N2	108.65 (18)	O1—C20—H20B	109.5
N1—C7—C8	108.36 (18)	H20A—C20—H20B	109.5
N2—C7—C8	142.99 (19)	O1—C20—H20C	109.5
C7—C8—C9	104.67 (19)	H20A—C20—H20C	109.5
C7—C8—C19	126.24 (19)	H20B—C20—H20C	109.5
C9—C8—C19	128.1 (2)	O4—C21—O3	124.0 (2)
C10—C9—C8	110.22 (19)	O4—C21—C9	123.7 (2)
C10—C9—C21	120.5 (2)	O3—C21—C9	112.17 (19)
C8—C9—C21	129.1 (2)	O3—C22—H22A	109.5
N1—C10—C9	105.65 (18)	O3—C22—H22B	109.5
N1—C10—C11	124.6 (2)	H22A—C22—H22B	109.5
C9—C10—C11	129.77 (19)	O3—C22—H22C	109.5
N3—C11—C10	177.5 (2)	H22A—C22—H22C	109.5
N2—C12—C13	109.47 (17)	H22B—C22—H22C	109.5
N2—C12—H12A	109.8	C7—N1—C10	111.09 (17)
C13—C12—H12A	109.8	C7—N1—C1	110.29 (17)
N2—C12—H12B	109.8	C10—N1—C1	138.55 (18)
C13—C12—H12B	109.8	C7—N2—C6	106.77 (16)
H12A—C12—H12B	108.2	C7—N2—C12	126.79 (18)
C14—C13—C18	119.0 (2)	C6—N2—C12	124.71 (17)
C14—C13—C12	120.0 (2)	C19—O1—C20	115.5 (2)
C18—C13—C12	121.1 (2)	C21—O3—C22	115.44 (19)
C13—C14—C15	120.6 (2)		
